# Implementation of the new S2e guideline “first-trimester diagnostics and therapy @ 11–13 + 6 weeks of pregnancy”: a survey in outpatient practices

**DOI:** 10.1007/s00404-025-08064-w

**Published:** 2025-05-27

**Authors:** J. Schipper, R. Menkhaus, A. Treier, V. Dütemeyer, A.L. Biermann, P. Hillemanns, C. von Kaisenberg, L. Brodowski

**Affiliations:** 1 Dr. Schmidt-Pich und Kollegen MVZ GmbH, Georgstraße 34, 30159 Hannover, Germany; 2 Department of Gynecology and Obstetrics, Hannover Medical School, Carl-Neuberg-Straße 1, 30625 Hannover, Germany; 3 Kinderwunschzentrum Minden, Simenonsplatz 17, 32423 Minden, Germany

**Keywords:** First-trimester screening, Guideline implementation, Prenatal diagnostics

## Abstract

**Purpose:**

The objective of this study was to ascertain the awareness of the guideline since its publication, its impact on outpatient approaches, implementation, and any alterations to daily practices.

**Methods:**

A questionnaire containing 36 items pertaining to first-trimester diagnostics and therapy was distributed to certificated gynecologists specializing in screening in Lower Saxony and East Westphalia. The questionnaire contained both multiple choice and open questions. The questions addressed information about the structure of the study population, the presentation of mandatory and optionally recommended settings for early structured fetal malformation diagnostics and echocardiography, the basic treatment regimens for first-trimester screening, and the utilization and management of non-invasive fetal test systems.

**Results:**

92.1% of the 38 respondents were familiar with the guideline at the time of the survey. The mandatory recommended structures are consistently displayed by 92.1–100% of participants, the remaining optional settings are displayed ranging from 7.9 to 55.3%. The majority of participants consider the combination of biochemistry and ultrasound markers as an indication for invasive diagnostics. 71.1% offer a non-invasive prenatal test in parallel with the first-trimester screening, while 65.8% of participants offer screening for preeclampsia. It was indicated by the participants that the publication of the guideline tended to have an impact on the outpatient approach to first-trimester screening. On average, the participants have modified their practice in first-trimester screening since the guideline was published.

**Conclusion:**

The recommendations outlined in the guideline have achieved a high level of awareness among most outpatient specialists, thereby exerting a notable influence on the formulation of treatment algorithms. In the contemporary context of patient-centered medicine and rapidly evolving healthcare, guidelines have emerged as a pivotal instrument for the effective aggregation of information.

**Supplementary Information:**

The online version contains supplementary material available at 10.1007/s00404-025-08064-w.

## Introduction

According to the definition of the Institute of Medicine (IOM), the guidelines are systematically developed decision-making aids to deal appropriately with specific health problems. The overarching objective of these guidelines is to improve the quality of patient care [1]. The Association of Scientific Medical Societies (AWMF) differentiates between three categories of guidelines. The first category, designated S1, comprises recommendations for action that are developed by an expert group in an informal consensus. The second category, designated S2, comprises guidelines with formal consensus-building and/or formal evidence research. The third category, designated S3, comprises guidelines with all elements of systematic development. It is becoming increasingly evident that a growing number of German physicians are coming to accept these guidelines, thus facilitating a significant contribution to the transfer of knowledge into practice [2, 3].

However, as the number of guidelines increases, so does the amount of information. The extensive scope of these guidelines and the numerous, detailed recommendations represent a substantial challenge for individual physicians. As the breadth of a field expands, the extent to which physicians need to possess comprehensive knowledge of guidelines concomitantly increases. In January 2024, the S2e guideline “First trimester diagnostics and therapy @ 11–13 + 6 weeks of pregnancy” was published [4].

The purpose of screening in the first trimester of pregnancy, at 11–13 + 6 weeks of gestation, is to identify risk factors that necessitate further diagnostic procedures and/or intervention at an early stage in pregnancy by a comprehensive evaluation of fetal biometry, the placenta, and amnion fluid. A significant component of the examination involves the search for malformations, checked by normal appearance of organs and structures, such as the head, brain, face, neck, thorax, heart, abdominal wall, gastrointestinal tract, extremities, spine, and genital. Other components are the risk calculation of developing preeclampsia and growth restriction, the screening for chromosomal disorders, particularly for trisomy 21, 13, and 18 and the investigation of other pregnancy problems. The utilization of the non-invasive prenatal testing (NIPT) is likewise addressed in the guideline [4]. The accelerated progression of sonographic, biochemical and molecular methodologies during the first-trimester screening has necessitated the formulation of proposals for a systematized and quality-assured approach, with the objective of providing patients with optimal counseling, diagnostics, and prevention. The guideline proposes a mandatory approach and an optional approach, while concomitantly endeavoring to furnish information on the composition of a customized pregnancy management. As outlined in the published guideline, in instances where the first trimester screening cannot be conducted in accordance with the stipulated standards, the patient should be referred to an appropriate facility. Additionally, the patient must be thoroughly informed about the purpose and protocol of the first trimester screening. This recommendation is categorized as the strongest level of recommendation, and a deviation is only permissible in the event of substantial medical justification [5, 6].

The aim of this study was to examine the awareness of the guideline since its publication, its impact on outpatient approaches, implementation, and any alterations to daily practices. Therefore, a questionnaire survey was conducted among outpatient colleagues in Lower Saxony and in East Westphalia. The survey solicited information on current practices, the perceived comprehensibility of the recommendations, any challenges arosing from them, and the feasibility of implementing the guideline.

## Methods

### Study design

This is a descriptive observational study. The study population comprised of physicians who were certified for first-trimester screening by either the Fetal Medicine Foundation (FMF) Germany or FMF United Kingdom (UK) at the time of the study and who were practicing in Lower Saxony or East Westphalia. The exclusion criteria included all physicians in these areas who did not perform first-trimester scans or who were not certificated.

### Data collection

In July 2024, a total of 141 physicians in Lower Saxony and East Westphalia were identified as having received certification for first-trimester screening from either the FMF Germany or FMF UK, and who conduct first-trimester screening independently.

Following the provision of information, written consent, and inclusion, a survey was dispatched via post. The eligible physicians who did not respond initially received a reminder email after 30 days, and a reminder phone call was made after an additional 8 weeks. The evaluation of the questionnaires was conducted anonymously. The participants were free to withdraw from the study at any time.

### Assessment instruments

The questionnaire was designed to assess the implementation of the new S2e guideline, entitled “First-trimester diagnostics and therapy @ 11–13 + 6 weeks of pregnancy”. The questionnaire comprised 36 items: 34 multiple choice questions, two closed questions with a purely categorical query on a 10-point Likert scale, and one open question for feedback on the guideline.

The questions addressed information about the structure of the study population, the presentation of mandatory and optionally recommended settings for early structured fetal malformation diagnostics and echocardiography, the basic treatment regimens for first-trimester screening and use and handling of non-invasive fetal test systems. The survey included inquiries regarding screening for preeclampsia and measurement of cervical length. In conclusion, the awareness of the guideline since its publication was queried, as was its impact on outpatient approaches and any alterations to daily practices. The questionnaire was developed in a manner consistent with extant studies examining the implementation of guidelines [7–9]. It was subjected to a rigorous validation process by five experts, who were tasked with ensuring its relevance and comprehensibility to the target respondents.

### Statistical analysis

The statistical analyses were performed using Graph Pad Prism 9 software (GraphPad Software Inc.). Means and standard deviation (SD) as well as numbers and percentages were calculated to present descriptive information. The determination of the data distribution was achieved through the utilization of the Shapiro–Wilk normality test.

### Ethics

Approval for this study was granted by the local ethics committee of Hannover Medical School, and it was conducted in accordance with the principles of the Declaration of Helsinki (approval number: 11468_BO_K_2024). The participants who completed the questionnaire were required to provide written informed consent.

## Results

### Structure of the study population

In total, 38 questionnaires were returned and included in the analysis (participation rate 26.9%). The general characteristics of the participating physicians are shown in Table 1. Most participants were certified by FMF Germany (57.9%) or FMF UK (42.1%). 5.3% of participants did not specify any certification, 7.9% of participants were certified for both FMF Germany and FMF UK. All carried out the first-trimester screening independently. The majority of the predominantly female participants (63.2%) performed the risk calculation independently using software (57.9%). Most of the respondents were between 51 and 60 years old (57.9%) and had between 21 and 30 years of professional experience (44.7%). The mean number of first-trimester screenings carried out by participants per month was between 11 and 20 (39.5%). It was found that 63.2% of practitioners had a standard operating procedure for carrying out the first-trimester screening.

**Table 1:** Structure of the study population

Characteristics	*N* (%)
Number of participants	38 (100)
Independent implementation of first-trimester screening	38 (100)
Certified by FMF UK	16 (42.1)
Certified by FMF Germany	22 (57.9)
Not specified	2 (5.3)
Certified by FMF UK and FMF Germany	3 (7.8)
Risk calculation process	
Independent risk calculation	22 (57.9)
Risk calculation by laboratory	10 (26.3)
Not specified	6 (15.8)
Sex	
Female examiners	24 (63.2)
Age group	
25–40 years of age	1 (2.6)
41–50 years of age	9 (23.7)
51–60 years of age	22 (57.9)
61–70 years of age	6 (15.8)
Professional experience	
0–10 years	0 (0)
11–20 years	11 (28.9)
21–30 years	17 (44.7)
> 30 years	10
Certified by DEGUM level	
Not certified by DEGUM	10 (26.3)
Level 1	9 (23.7)
Level 2	19 (50)
Level 2 Kursleiter	0 (0)
Level 3	0 (0)
Number of first-trimester screenings performed per month	
0–10	12 (31.6)
11–20	15 (39.5)
21–30	4 (10.5)
31–40	5 (13.2)
> 40	2 (5.3)
Digital documentation of the first-trimester screening	
Viewpoint	17 (44.7)
Astralia	6 (15.8)
FMF UK software	3 (7.9)
FMF Germany software	11 (28.9)
No digital documentation	6 (15.8)
Existing standard operating procedure for first-trimester screening	24 (63.2)

### Presentation of the mandatory and optionally recommended settings for early structured malformation diagnostics and fetal echocardiography

The mandatory recommended structures are consistently displayed by 92.1–100% of all participants. Although the scanning of the position of the heart, the contour and the 4-chamber view is mandatory, this is not always performed optionally by 7.9% of participants.

In relation to the optional recommended structures of the heart, the cardiac axis is most frequently always displayed with 84.2%. The remaining optional settings are scanned less frequently, ranging from 7.9 to 55.3%. The exclusion of an aberrant subclavian artery is most frequently never done (44.7%).

The nasal bone is consistently displayed in 92.1% of cases, despite its optional recommendation. Conversely, the ductus venosus flow and tricuspid flow are scanned less frequently, at 55.3 and 50%, respectively (Table 2).

**Table 2:** (a) Presentation of the mandatory recommended settings for early structured malformation diagnostics. (b) Presentation of the optionally recommended settings for early structured malformation diagnostics. (c) Presentation of the mandatory and optionally recommended settings for early structured fetal echocardiography

Structure	Is always scanned, *N* (%)	Is optional scanned, *N* (%)	Is never scanned, *N* (%)
(a)			
Nuchal translucency	37(97.4)	1 (2.6)	0 (0)
Skull and brain with calotte, falx cerebri and choroid plexus	36 (94.7)	1 (2.6)	1 (2.6)
Profile	37 (97.4)	1 (2.6)	1 (2.6)
Heart position, contour, 4-chamber view	35 (92.1)	3 (7.9)	0
Abdomen, stomach, abdominal wall	37 (97.4)	1 (2.6)	0 (0)0
Extremities: arms	38 (100)	0 (0)	0 (0)
Extremities: legs	38 (100)	0 (0)	0 (0)
Bladder	38 (100)	0 (0)	0 (0)
Gemini: chorionicity and amniocity	37 (97.4)	1 (2.6)	0 (0)
(b)			
Nasal bone	35 (92.1)	3 (7.9)	0 (0)
Ductus venosus flow	21 (55.3)	11 (28.9)	6 (15.8)
Tricuspid flow	19 (50)	10 (26.3)	9 (23.7)
(c)			
Mandatory recommended			
Heart position	36 (94.7)	2 (5.3)	0 (0)
4-chamber view	36 (94.7)	2 (5.3)	0 (0)
Optionally recommended			
Cardiac axis	32 (84.2)	5 (13.2)	1 (2.6)
Right ventricular outflow tract	18 (47.4)	16 (42.1)	4 (10.5)
Left ventricular outflow tract	17 (44.7)	16 (42.1)	5 (13.2)
3-vessel trachea view	21 (55.3)	12 (31.6)	5 (13.2)
Exclusion of aberrant subclavian artery	3 (7.9)	18 (47.4)	17 (44.7)

### Basic treatment regimens for first-trimester screening

The correction of the due date by measured crown-rump length was carried out in 21.1% of all participants, with the majority (76.3%) relying on the difference to the originally expected date of delivery for this adjustment.

Furthermore, 36.8% of all participants offer the first-trimester screening without laboratory services. 63.2% offer the screening without measuring ß-HCG and PAPP-A, while 26.3% of screenings involved the performance of laboratory services depending on the results of the first-trimester screening risk calculation relying on anatomic structures.

Nearly half of the participants perform amniocentesis/chorionic villus sampling by themselves. In this case, the majority of the participants (79%) consider the combination of biochemistry and ultrasound markers as an indication for invasive diagnostics. The majority of respondents define a measurement above 3 mm of the nuchal translucency as the limit value for performing amniocentesis/chorionic villus sampling (42.1%) (Table 3).

**Table 3:** Basic treatment regimens for first trimester screening

Characteristics	*N* (%)
Correction of the due date by measured crown-rump length	
No	1 (2.63)
Always	8 (21.1)
Depending on the distance to the originally expected date of delivery	29 (76.32)
Conducting first-trimester screening with ß-HCG and PAPP-A	
Yes	24 (63.16)
No	4 (10.53)
Optional	10 (26.32)
Offer of first-trimester screening without laboratory services	
Yes	14 (36.84)
No	11 (28.95)
Optional	13 (34.21)
Does the performance of laboratory services depend on performed risk calculation of first-trimester screening	
Yes	10 (26.32)
No	15 (39.47)
Optional	13 (34.21)
Performance of an amniocentesis/chorionic villus sampling autonomously	
Yes	17 (44.74)
Dependence of the decision for amniocentesis/chorionic villus sampling	
Nuchal translucency measurement / limits of nuchal translucency	20 (52.63)
Biochemistry (ß- HCG and PAPP A)	7 (18.42)
Combination of first-trimester screening and biochemistry	30 (78.95)
Limit value of the nuchal translucency measurement for performing amniocentesis/chorionic villus sampling	
> 2.5 mm	9 (23.68)
> 3.0 mm	16 (42.11)
> 3.5 mm	7 (18.42)
No value	4 (10.53)

### Use and handling of non-invasive fetal test systems

Most participants offer a non-invasive prenatal test (NIPT) in parallel with the first-trimester screening (71.1%), while sequential testing is offered less frequently, with a NIPT first, followed by the first-trimester screening later (15.8%). Only 39.5% of participants offer the NIPT without first-trimester screening. The majority of these participants offer a package of tests for trisomy 21, 13 and 18, as well as sex chromosomes and fetal gender (63.2%). Non-invasive fetal test systems for special diseases such as Di-George syndrome are rarely used (15.8%) (Table 4).

**Table 4:** Use and handling of non-invasive fetal test systems

Characteristics	*N* (%)
Offer of first-trimester screening parallel to non-invasive prenatal test	
Yes	27 (71.05)
No	0 (0)
Optional	11 (28.95)
Offer of non-invasive prenatal testing in a two-stage procedure before first trimester screening	
Yes	6 (15.79)
Offer of non-invasive prenatal testing from a certain threshold after completion of the first trimester screening	
> 1:1000	10 (26.32)
> 1:500	11(28.95)
> 1:100	11(28.95)
No	6 (15.79)
Offer of non-invasive prenatal testing after informed consent without performing first-trimester screening	
Yes	15 (39.47)
The following question pertains to the provision of a non-invasive prenatal test	
Trisomy 21, 13 and 18	8 (21.1)
Trisomy 21, 13 and 18, monosomy and fetal sex	24 (63.16)
Other diseases such as Di-George syndrome	6 (15.79)

### Screening for preeclampsia and measurement of cervical length

65.8% of participants offer screening for preeclampsia, with 20% opting for the measurement of the uterine arteries and 80% selecting the combination of uterine artery measurement and PLGF measurement in the pregnant woman’s serum. 72% of practitioners set the cut-off for prophylactic treatment with 150 mg of ASS daily, based on an individually calculated risk of greater than 1:100.

Furthermore, it is notable that 65.8% of the practitioners incorporate cervical length measurement as a component of the first trimester examination (Table 5).

**Table 5:** Screening for preeclampsia and measurement of cervical length

Characteristics	*N* (%)
Offer screening for preeclampsia	
Yes	25 (65.79)
Conducting preeclampsia screening with	
Measurement of Aa. uterinae	5 (20)
Measurement of Aa. uterinae and PLGF	20 (80)
Recommendation for prophylaxis with ASS 150 mg	
Risk for PE > 1:100	18 (72)
Risk of PE > 1:150	4 (16)
Different cut off	3 (12)
Measurement of cervical length during first-trimester screening	
Yes	25 (65.79)

### Implementation of the new S2e guideline “First-trimester diagnostics and therapy @ 11–13 + 6 weeks of pregnancy”

**Fig. 1:**
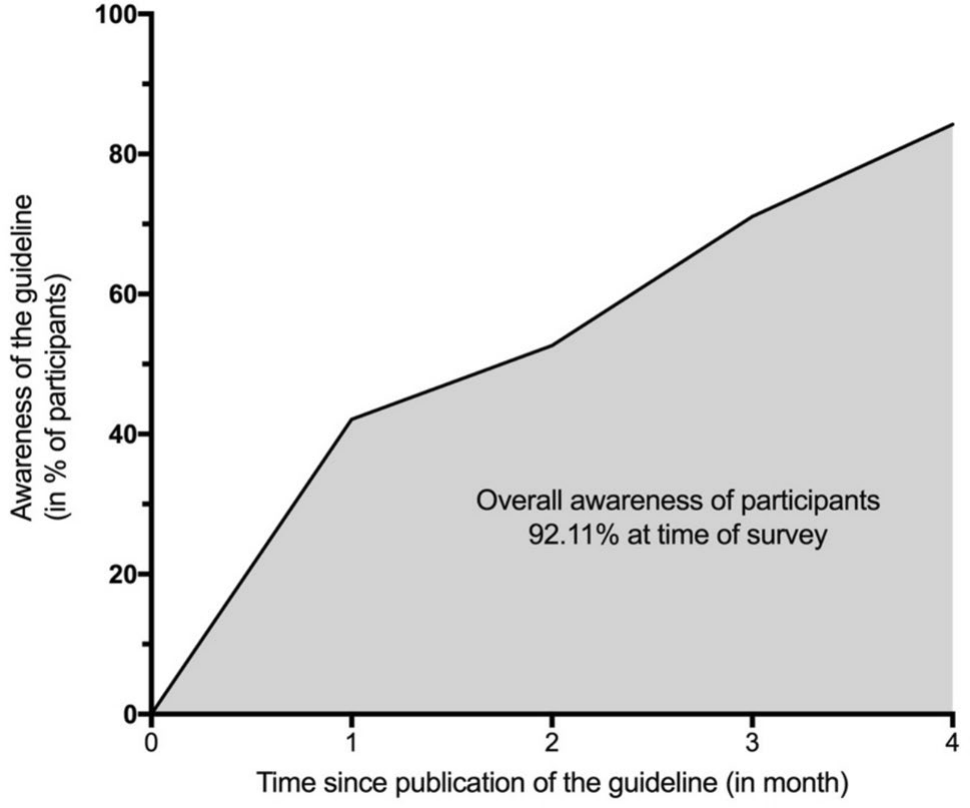
Awareness of the guideline depending on the time of publication

92.1% of respondents were familiar with the guideline at the time of the survey. Approximately 50% of the collective had already acquainted themselves with the guideline 1 month after its publication, and after 3 months, the awareness increased to approximately 75% (Fig. 1).

**Fig. 2:**
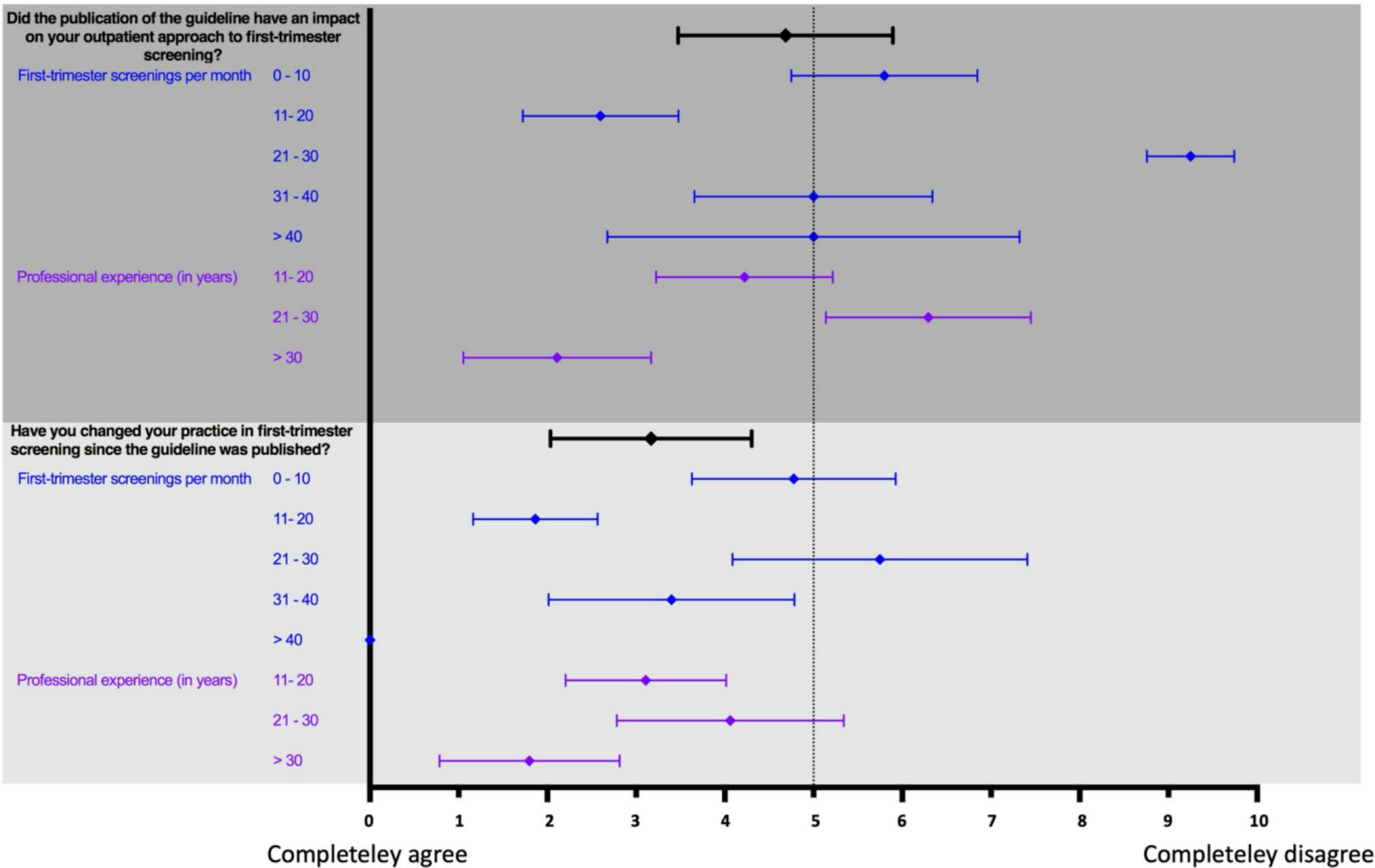
Implementation of the new S2e guideline “First-trimester diagnostics and therapy @ 11–13 + 6 weeks of pregnancy”

Overall, the participants indicated that the publication of the guideline tended to have an impact on the outpatient approach to first-trimester screening. Regarding the participants’ professional experience, the influence of the guideline was most marked among those with 11–20 years of professional experience and over 30 years. Furthermore, the number of screenings performed per month was found to be a factor in the perceived impact of the guideline on work practices, with those performing 10–20 screenings per month indicating the most substantial impact. On average, the participants have modified their practice in first-trimester screening since the guideline was published. This change was particularly evident among practitioners who conduct a lower or high volume of screenings and those with the most extensive professional experience (Fig. 2).

## Discussion

The implementation of guidelines in practice constitutes the process of integrating scientifically based recommendations or standards into daily work routines. The process is complex and challenging due to its multifaceted nature. It entails the translation of theoretical concepts into practical applications, while concomitantly encompassing the consideration of extant structures, resources and the cooperation of various actors. The implementation of these guidelines necessitates meticulous planning, ongoing training, and close collaboration among all stakeholders. To achieve the desired improvement, it is essential that all relevant parties understand the guidelines and acknowledge their importance in practice.

The enhancement of communication and analytical instruments has resulted in the dissemination of a considerable number of clinical findings on a daily basis. This has given rise to a marked discrepancy between the substantial public health knowledge generated through clinical research and its practical application in clinical settings [10].The data presented here demonstrate that 92.1% of the respondents were familiar with the guideline at the time of the survey. However, it is imperative to note that certain recommendations of the highest priority are not being adequately implemented.

The mandatory recommended structures are consistently displayed by 92.1–100% of participants, while the remaining optional settings are displayed ranging from 7.9 to 55.3%. The four-chamber view is mandatory in accordance with the guideline. However, this view is not always performed by 7.9% of participants. The guideline signifies the inaugural framework in Germany for first-trimester screening. The FMF has established recommendations for scanning protocols and structural assessments in both the UK and Germany. However, there is currently no official verification process for these structures.

Since the beginning of this century, the prenatal non-invasive screening has been offered to pregnant women in Europe based on risk calculation programs developed by the FMF UK and FMF Germany. These programs have since spread worldwide. [11]. Although first-trimester screening in Germany is at the patient’s expense, it is highly acceptable to pregnant women (up to 50%) [12].

Since July 2022, statutory health insurance companies in Germany have covered the costs of molecular genetic NIPT for trisomy 13, 18 and 21.

Despite not being formally recognized as a screening test in German healthcare protocols, the growing acceptance of NIPT has led to a steady decline in the utilization of first-trimester screenings since 2018, as evidenced by data from the leading prenatal laboratory in Germany, Amedes Genetics Lab [11, 13]. Our findings demonstrate that most participants offer a NIPT in conjunction with first-trimester screening (71. 1%), while sequential testing is offered less frequently, with a NIPT first, followed by the first-trimester screening later (15.8%). Only 39.5% of the participants offer the NIPT without first-trimester screening. A function of the first-trimester screening is to serve as a preliminary assessment, determining whether further NIPT is advised [14, 15]. The indication for NIPT primarily pertains to patients without fetal malformations and an intermediate risk for trisomy 21 [16, 17]. First-trimester screening is not a component of standard prenatal care in Germany, and the utilization of NIPT remains largely unrestricted, with the exception of specific guidelines issued by the German Society for Ultrasound in Medicine (DEGUM). The German Federal Joint Committee (Gemeinsamer Bundesausschuss, G-BA) has stipulated that NIPT can be performed at patients’ request without prior risk stratification and regardless of maternal age. Since January 2022 the German legal framework has made coverage of the cost of NIPT possible for specific indications [18]. The implementation of NIPT has presented a significant challenge to proponents of traditional first- trimester screening due to the enhanced screening performance of NIPT for trisomy 21, the ongoing decline in NIPT costs, and the expansion of its diagnostic capabilities in recent years [18].

Preeclampsia has been shown to be associated with significant morbidity and mortality for maternal and fetal health [19]. 65.8% of participants offer screening for preeclampsia, and 72% of practitioners set the cut-off for prophylactic treatment with 150 mg of ASS daily, based on an individually calculated risk of greater than 1:100. This corresponds to the specification laid down in the guideline. Preeclampsia screening is not a mandatory component of the first trimester screening in Germany, nor is it incorporated within the German statutory maternity guidelines. Screening for preeclampsia results in additional costs and time expenditure for the patients and practitioners contributing to the low rate of acceptance. In contrast, in the United Kingdom, preeclampsia screening is mandatory during pregnancy and covered by the national health insurance system. In the German healthcare system, the cost of screening for preterm preeclampsia versus routine screening based on maternal characteristics is 14 euros per person. This is not considered cost-effective [20]. However, it was shown that taking aspirin in high-risk patients from the first trimester until 36 weeks gestation prevents 80% of cases with preeclampsia before 32 weeks gestation [21]. In addition, further cost-effectiveness studies are needed covering longer period of lifetime.

In the first trimester guideline and in the international literature, the cut-off for invasive testing regarding the nuchal translucency is 3 mm, at the latest at 3.5 mm [4, 22]. The majority of the participants in our study define a measurement of 3 mm as the limit value of an isolated nuchal translucency measurement for performing amniocentesis/chorionic villus sampling (42.1%). Only half of the participants in this study offer invasive testing. Additional training options and learning concepts should be implemented for prenatal scanning to be able to offer invasive diagnostics more widely.

The present study has some limitations. The survey response rate was considered low with 27%. This is probably due to the time demands of resuming clinical work on a daily basis, which may have prevented the respondents from allocating sufficient time to the survey. The survey was conducted exclusively in Lower Saxony and East Westphalia which may have led to a selection bias. It is conceivable that the participants in the study had already familiarized themselves with the first-trimester guidelines before inclusion, and thus felt more prepared to participate.

It is imperative that examiners possess the requisite skills and knowledge to facilitate the effective execution of first-trimester screening procedures. The findings of this study demonstrate that the majority of respondents exhibit familiarity with the guideline and consistently perform recommended structures. The results further indicate that the publication of the guideline tend to have an impact on the outpatient approach to first-trimester screening. On average, participants have modified their practice in first-trimester screening since the guideline was published.

The recommendations outlined in the guideline have achieved a high level of awareness among most outpatient specialists, thereby exerting a notable influence on the formulation of treatment algorithms. In the contemporary context of patient-centered medicine and rapidly evolving healthcare, guidelines have emerged as a pivotal instrument for the effective aggregation of information.

## Supplementary Information

Below is the link to the electronic supplementary material.

Supplementary file1 (PDF 89 KB)

## Data Availability

Raw data were generated at the Department of Obstetrics and Gynecology of Hannover Medical School (MHH). The datasets used and analyzed during the current study are available from the corresponding author upon reasonable request.
