# Supplementary material for: Implementation of the new S2e guideline “first-trimester diagnostics and therapy @ 11–13 + 6 weeks of pregnancy”: a survey in outpatient practices
Source: Arch Gynecol Obstet. 2025 May 27;312(3):755–64. doi: 10.1007/s00404-025-08064-w (PMC12374909; doi:10.1007/s00404-025-08064-w)
Supplement: Supplementary file 1 — Supplementary file1 (PDF 89 KB) [file 404_2025_8064_MOESM1_ESM.pdf]

Bitte kreuzen Sie zutreffendes an, Mehrfachauswahl möglich:

☐ Ich führe das Ersttrimesterscreening selbstständig durch
☐ Ich bin nach FMF UK zertifiziert
☐ Ich bin nach FMF Deutschland zertifiziert
☐ Ich führe die Risikoberechnung selbstständig durch
☐ Die Risikoberechnung wird durch das Labor durchgeführt

Welchem Geschlecht fühlen Sie sich zugehörig?
☐ männlich
☐ weiblich
☐ divers

Welcher Altersklasse gehören Sie an?
☐ 25-40 Jahre
☐ 40-50 Jahre
☐ 50-60 Jahre
☐ 60-70 Jahre

Wie viele Jahre Berufserfahrung haben Sie?
☐ 0-10 Jahre
☐ 10-20 Jahre
☐ 20-30 Jahre
☐ > 30 Jahre

Haben Sie eine DEGUM- Stufe?
☐ Nein
☐ DEGUM Stufe 1
☐ DEGUM Stufe 2
☐ DEGUM Stufe 2 Stufenleiter
☐ DEGUM Stufe 3

Wie viele Ersttrimesterscreenings führen Sie ungefähr in einem Monat durch?
☐ 0-10
☐ 10-20
☐ 20-30
☐ 30-40
☐ >40

Ist in Ihrer Praxis eine Dokumentationsmöglichkeit vorhanden?
☐ Viewpoint
☐ Astralia
☐ FMF UK Software
☐ FMF Deutschland Software
☐ Nein

Ist in Ihrer Praxis ein Handlungsablauf/ QM- Dokument vorhanden zum Ersttrimesterscreening?

☐ Ja

☐ Nein

Ersttrimesterscreening:

Mit welchen folgenden Parametern/Messwerten führen Sie das Ersttrimesterscreening durch (Mehrfachantworten möglich)?

Struktur	Stelle ich immer dar	Stelle ich optional dar	Stelle ich nie dar
Nackentransparenz			
Nasenbein			
Ductus venosus			
Trikuspidalklappe Flow			
Schädel und Gehirn mit Kalotte, Falx cerebri und Plexus choroidei			
Profil			
Herz mit Lage, Kontur, Vierkammerblick			
Abdomen mit Magen und Bauchwand			
Arme			
Beine			
Harnblase			
Gemini: Chorionizität, Amnionizität			

Was stellen Sie im Rahmen der fetalen Echokardiographie im Ersttrimesterscreening dar (Mehrfachantworten möglich)?

Struktur	Stelle ich dar	Stelle ich optional dar	Stelle ich nie dar
Lage des Herzens			
Herzachse			
Vierkammerblick			
Rechtsventrikulärer Ausflusstrakt			
Linksventrikulärer Ausflusstrakt			
Drei- Gefäß Trachea Blick mit Aorten und Ductusbogen			
Ausschluss Aberrante Rechte A. subclavia			

Bei der von Ihnen erhobenen Scheitelsteißlänge erfolgt eine Korrektur des Entbindungstermins?

☐ Nein
☐ Ja, immer
☐ Ja, abhängig vom Abstand zum Entbindungstermin

Bieten Sie das Ersttrimesterscreening mit den Laborwerten: ß- HCG und PAPP- A an?

☐ Ja
☐ Nein
☐ Optional

Bieten Sie das Ersttrimesterscreening mit dem Nicht- invasiven Pränataltest an?

☐ Ja
☐ Nein
☐ Optional

Bieten Sie das Ersttrimesterscreening ohne Laborleistungen an?

☐ Ja
☐ Nein
☐ Optional

Hängt die Durchführung von Laborleistungen an der Risikokalkulation des durchgeführten Ersttrimesterscrenning ab?

☐ Ja
☐ Nein
☐ Optional

Bieten Sie den Nicht- invasiven Pränataltest in einem zweizeitigen Vorgehen an, zB in der 10. SSW Durchführung des NIPT und ab der 12. SSW Durchführung der Ultraschalluntersuchung?

☐ Ja
☐ Nein

Bieten Sie den Nicht- invasiven Pränataltest ab einem bestimmten Grenzwert nach Durchführung des Erstrimesterscreenings an?

☐ Ja
- ☐< 1:1000
- ☐< 1:500
- ☐<1:100

☐ Nein

Bieten Sie den Nicht- invasiven Pränataltest nach Aufklärung ohne Durchführung des Ersttrimesterscreening an (zB von der Patientin nicht gewünscht)?

☐ Ja
☐ Nein

Bieten Sie den Nicht- invasiven Pränataltest nur für Trisomie 21, 13 und 18 oder auch in anderen Kombinationen an (unabhängig von der Übernahme der Krankenkasse)?

☐ Trisomie 21, 13 und 18
☐ Trisomie 21, 13 und 18, Monosomie und Geschlecht
☐ andere Erkrankungen wie zB Di- George Syndrom

Führen Sie (falls notwendig) eine Amniozentese/ Chorionzottenbiopsie selbstständig durch?
☐ Ja
☐ Nein

Führen Sie die Entscheidung zur Amniozentese/ Chorionzottenbiopsie abhängig von der aus?
☐ Nackentransparenzmessung / Grenzwerte der Nackentransparenz
☐ Biochemie (ß- HCG und PAPP A)
☐ Kombination Ersttrimesterscreening und Biochemie

Ab welchen Grenzwert der Nackenfaltenmessung raten Sie Ihren Patientinnen zur Durchführung einer Amniozentese/ Chorionzottenbiopsie?
☐ NT > 2,5
☐ NT > 3,0
☐ NT > 3,5
☐ NT ______
☐ gar nicht

Präeklampsie- Screening:

Führen Sie das Präeklampsie- Screening durch?
☐ Ja
☐ Nein

Führen Sie das Präeklampsie Screening durch mit
☐ A. uterina Messung
☐ A. uterina Messung und PLGF Bestimmung

Beim durchgeführten Präeklampsie- Screening, ab welchen Grenzwerten verordnen Sie Aspirin 150 mg 0-0-0-1 zur Prophylaxe?
☐ < 1:100
☐ <1:150
☐ anderer Grenzwert _________
☐ gar nicht

Zervixlängenmessung:

Führen Sie eine Zervixlängenmessung im Risikokollektiv im Ersttrimesterscreening durch?
☐Ja
☐Nein

Bewertung:

Ist Ihnen die S2e Leitlinie „Ersttrimester Diagnostik und Therapie @ 11-13+6 Schwangerschaftswochen“ bekannt?

☐Ja
☐Nein

Wenn ja, seit wann ist Ihnen die Leitlinie bekannt?

____

Hatte die Veröffentlichung der Leitlinie einen Einfluss auf Ihr ambulantes Vorgehen bezüglich des Ersttrimesterscreenings?

Trifft voll zu -- Trifft gar nicht zu

Haben Sie Ihren Handlungsablauf bezüglich des Ersttrimesterscreenings nach Erscheinen der Leitlinie geändert?

Trifft voll zu -- Trifft gar nicht zu

Fehlt Ihnen etwas in der Leitlinie?
Freitext: ____

____

____
